# Clinical trial recruitment of people who speak languages other than English: a Children’s Oncology Group report

**DOI:** 10.1093/jncics/pkae047

**Published:** 2024-06-18

**Authors:** Melissa P Beauchemin, Maria Ortega, Sheila J Santacroce, Joanna M Robles, Jenny Ruiz, Anurekha G Hall, Justine M Kahn, Cecilia Fu, Manuela Orjuela-Grimm, Grace C Hillyer, Samrawit Solomon, Wendy Pelletier, Raul Montiel-Esparza, Lindsay J Blazin, Cassie Kline, Alix E Seif, Paula Aristizabal, Lena E Winestone, Maria C Velez

**Affiliations:** Division of Research and Scholarship, Columbia University School of Nursing, New York, NY, USA; Herbert Irving Comprehensive Cancer Center, Columbia University Irving Medical Center, New York, NY, USA; Children’s Hospital of Philadelphia, Division of Oncology, Philadelphia, PA, USA; School of Nursing and Linberger Comprehensive Cancer Center, University of North Carolina at Chapel Hill, Chapel Hill, NC, USA; Pediatric Oncology, Wake Forest University Health Sciences, Winston Salem, NC, USA; University of Pittsburgh School of Medicine, Pittsburgh, PA, USA; University of Washington School of Medicine and Seattle Children’s Hospital, Seattle, WA, USA; Herbert Irving Comprehensive Cancer Center, Columbia University Irving Medical Center, New York, NY, USA; Pediatric Oncology, Columbia University Irving Medical Center, New York City, NY, USA; Keck School of Medicine, Children’s Hospital of Los Angeles, Los Angeles, CA, USA; Herbert Irving Comprehensive Cancer Center, Columbia University Irving Medical Center, New York, NY, USA; Pediatric Oncology, Columbia University Irving Medical Center, New York City, NY, USA; Herbert Irving Comprehensive Cancer Center, Columbia University Irving Medical Center, New York, NY, USA; Division of Research and Scholarship, Columbia University School of Nursing, New York, NY, USA; University of Calgary, Calgary, AB, Canada; Department of Pediatrics, Stanford Medicine, Palo Alto, CA, USA; Division of Oncology, Riley Children’s Hospital, Indianapolis, IN, USA; Children’s Hospital of Philadelphia, Division of Oncology, Philadelphia, PA, USA; Children’s Hospital of Philadelphia, Division of Oncology, Philadelphia, PA, USA; Division of Pediatric Hematology/Oncology, Department of Pediatrics, University of California San Diego/Rady Children’s Hospital San Diego and Moores Cancer Center, La Jolla, CA, USA; Division of Allergy, Immunology & BMT, University of California San Francisco Benioff Children’s Hospitals, San Francisco, CA, USA; Children’s Hospital New Orleans/Louisiana State University Health Sciences Center, New Orleans, LA, USA

## Abstract

**Background:**

Persons who speak languages other than English are underrepresented in clinical trials, likely in part because of inadequate multilevel resources. We conducted a survey of institutions affiliated with the Children’s Oncology Group (COG) to characterize current research recruitment practices and resources regarding translation and interpretation services.

**Methods:**

In October 2022, a 20-item survey was distributed electronically to institutions affiliated with COG to assess consent practices and resources for recruiting participants who speak languages other than English to COG trials. Descriptive statistics were used to summarize responses; responses were compared by institution size and type as well as respondent role.

**Results:**

The survey was sent to 230 institutions, and the response rate was 60% (n = 139). In total, 60% (n = 83) of those respondents had access to short-form consent forms. Full consent form translation was required at 50% of institutions, and 12% of institutional review boards restricted use of centrally translated consent forms. Forty-six percent (n = 64) of institutions reported insufficient funding to support translation costs; 19% (n = 26) had access to no-cost translation services. Forty-four percent (n = 61) were required to use in-person interpreters for consent discussions; the most frequently cited barrier (56%) to obtaining consent was lack of available in-person interpreters. Forty-seven percent (n = 65) reported that recruiting persons who speak languages other than English to clinical trials was somewhat or very difficult.

**Conclusions:**

Institutions affiliated with COG face resource-specific challenges that impede recruitment of participants who speak languages other than English for clinical trials. These findings indicate an urgent need to identify strategies aimed at reducing recruitment barriers to ensure equitable access to clinical trials.

In 2019, nearly 68 million persons in the United States reported speaking a language other than English at home, almost half of whom reported speaking English less than “very well” (1,2) Across the United States, the most commonly spoken languages are Spanish, Chinese (including Cantonese, Mandarin, and other dialects), French, Tagalog, and Vietnamese (1). The linguistic diversity of the US population is increasing; however, current system-level resources and practices hinder progress toward inclusive and effective health care for persons who speak languages other than English. Patients who speak languages other than English experience a breadth of health-care inequities, including underenrollment in clinical trials, decreased access to primary care, increased utilization of emergency care, and a higher incidence of treatment-related adverse events (3-6). Individuals who speak languages other than English express decreased satisfaction with health care, decreased understanding of their medical problems, increased medical complications, and worse quality of goals for care discussions (7-10).

Cancer clinical trials offer patients access to the newest technologies and treatments and are critical to advancing therapeutic options and improving clinical outcomes. Appropriate representation of minoritized and underrepresented populations in clinical research, including persons who speak languages other than English, is necessary to ensure equitable access to novel treatments and generalizable research findings (11). In May 2023, the National Institutes of Health Clinical Trial Diversity Act (12) was passed to enhance the inclusion of women, racially and ethnically diverse individuals, and people of all ages in National Institutes of Health–funded research, building on prior legislation initiated in 1994 (13), but underrepresentation of racial and ethnic minority populations and individuals of low-socioeconomic status persists (14-16). Reasons for disparities in clinical trial participation are multilevel (17), and structural and system-level barriers, such as rigid eligibility requirements, lack of trial availability, and resources required to conduct clinical trials, are commonly cited reasons for lower enrollment rates. In addition, economic costs and financial barriers, mistrust of the health-care system, limited health literacy, provider lack of awareness of clinical trials, or provider-patient language discordance may hinder patients’ access to and inclusion in clinical trials (18,19). If offered, however, most patients will agree to participate in cancer clinical trials (20).

Clinical trial participation among diverse patients representing the US population is a high priority for the National Cancer Institute (NCI)–funded Children’s Oncology Group (COG) (21). Though clinical trial participation has historically been higher in pediatric oncology than in adult oncology, lower participation rates have been reported among Hispanic patients and those who speak languages other than English, especially as pediatric patient grows older (22-24). Currently, the capacity of COG-affiliated institutions to obtain parental permission and consent from persons who speak languages other than English for treatment or supportive care clinical trials is not known. The purpose of this study was to characterize current research recruitment practices and resources regarding translation and interpretation for enrollment in COG clinical trials.

## Methods

A cross-sectional survey was distributed in October 2022 using Research Electronic Data Capture to principal investigators and lead clinical research associates at COG-affiliated institutions (25). Surveys are frequently administered to this group of individuals, who are responsible for COG clinical trials at 230 unique institutions across the United States, Canada, and Australia.

### Survey development

The survey was developed by the Language Equity Working Group within the COG Diversity and Health Disparities Committee (26). The working group designed a 20-question survey (Supplementary Methods, available online) to assess site-level characteristics, research recruitment practices, resources, and regulatory requirements for gaining consent from individuals who speak languages other than English to clinical trials, including use of interpreter services and short-form consent documents, an alternative to using a translated study consent form intended for unexpected enrollment of someone speaking a language other than English (27). To guide development of the survey, given the complexities and changing landscape of terminology and definitions regarding language, literacy, and inclusion, we developed a list of definitions with the understanding that preferred terminology regarding language equity is evolving (Supplementary Table 1, available online) (2,28-30). This study was approved by the Columbia University Institutional Review Board (IRB No. AAAU1679).

### Study participants and procedures

The survey was distributed to all COG principal investigators and lead clinical research associates by the central COG Communications office, requesting 1 response per institution. After a brief description of the survey, the respondent was prompted to click forward, signaling their consent to study participation. Upon request, a copy of the consent was emailed to the respondent. Respondents were able to save and return to complete their survey. No compensation was provided to study participation.

### Data analysis

The data were exported, and duplicate site responses were removed, prioritizing the COG principal investigator response. Descriptive statistics were used to report the respondent’s individual and institutional characteristics as well as survey item responses in aggregate using frequencies and ranges. We compared responses by respondent-level and institutional-level characteristics—specifically, we categorized institution size (≥100 vs <100 new pediatric cancer cases per year), type (academic, nonacademic), and respondent role (physician, nonphysician). We compared responses within each category using χ^2^ tests; *P* < .05 was considered statistically significant. We also conducted a sensitivity analysis to explore whether the results would differ if the non-COG principal investigator respondent were prioritized.

## Results

### Participant characteristics

**Figure 1.**
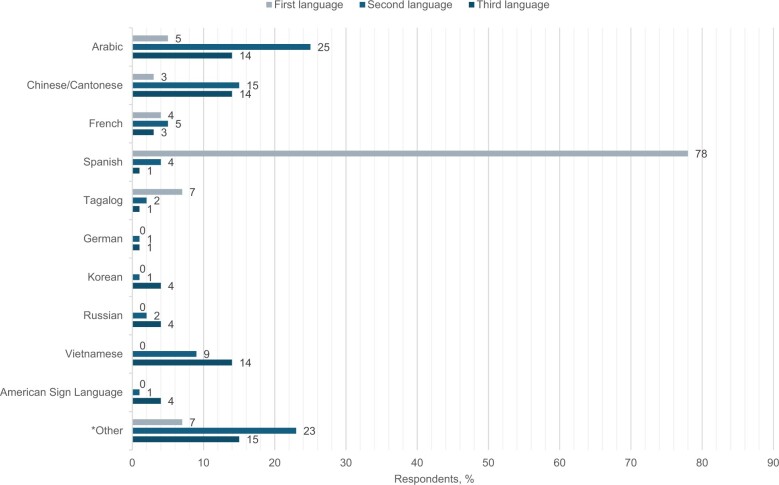
Ranking of languages other than English spoken by the patient population, by respondent. ^a^ “Other” includes Cambodian, Hindi, Hmong, Indigenous Cree, Creole, Karen, Kurdish, Urdu, and unspecified/not reported (n = 4).

The survey was sent to 230 COG institutions. We received 151 responses, but 12 were removed as duplicates at the site level. Overall, the institutional response rate was 60% (n = 139); 72% (n = 100) of respondents were from academic medical centers, and 71% (n = 99) were from small to medium-sized institutions seeing fewer than 100 new pediatric cancer cases each year (Table 1). Most respondents were located in the United States (n = 118 [85%]); 9% (n = 13) were in Canada, and 5% (n = 7) were in Australia. The respondent was most often a physician (n = 79 [57%]); 26% (n = 36) were clinical research associates, 9% (n = 12) were research nurses, and 8% (n = 11) were NCI Community Oncology Research Program administrators. Participants ranked the top 3 languages other than English most frequently spoken at their institution. By country, the most common language other than English spoken was Spanish in the United States, French in Canada, and Chinese in Australia (Figure 1). Health literacy screening for parents or patients 18 years of age or older was conducted at 24% (n = 33) of sites.

**Table 1.** Characteristics of responding Children’s Oncology Group institutions (N = 139)

Characteristic	No. (%)
Size of institution	
<100 new pediatric cancer cases/y	99 (71)
≥100 new pediatric cancer cases/y	40 (29)
Type of institution	
Academic medical center	100 (72)
Nonacademic community or public hospital	23 (17)
Nonacademic private hospital	12 (9)
Military hospital	2 (1)
Other	2 (1)
Location^a^	
United States	118 (85)
Canada	13 (9)
Australia	7 (5)
Affiliated with Clinical Translational Science Award	
Yes	20 (14)
No	54 (39)
Don’t know	65 (47)
Respondent discipline/role^a^	
Physician	79 (57)
Clinical research associate	36 (26)
Nurse	12 (9)
National Cancer Institute Community Oncology Research Program administrator	11 (8)
Most common language other than English spoken by patients, by country of respondent site (see also Figure 1)^a^	
United States—Spanish	107/117 (91)
Canada—French	6/13 (46)
Australia—Chinese	3/6 (50)
Bilingual/multilingual clinicians	
None	24 (17)
1% to <10%	38 (27)
10%-25%	46 (33)
>25%	25 (18)
If ≥1%, certified multilingual clinicians	
Yes	32/109 (29)
Health literacy screening for parents or patients ≥18 y of age	
Yes	33 (24)

a May not sum to total due to missing response (n = 1).

### Consent documents and regulatory requirements

**Figure 2.**
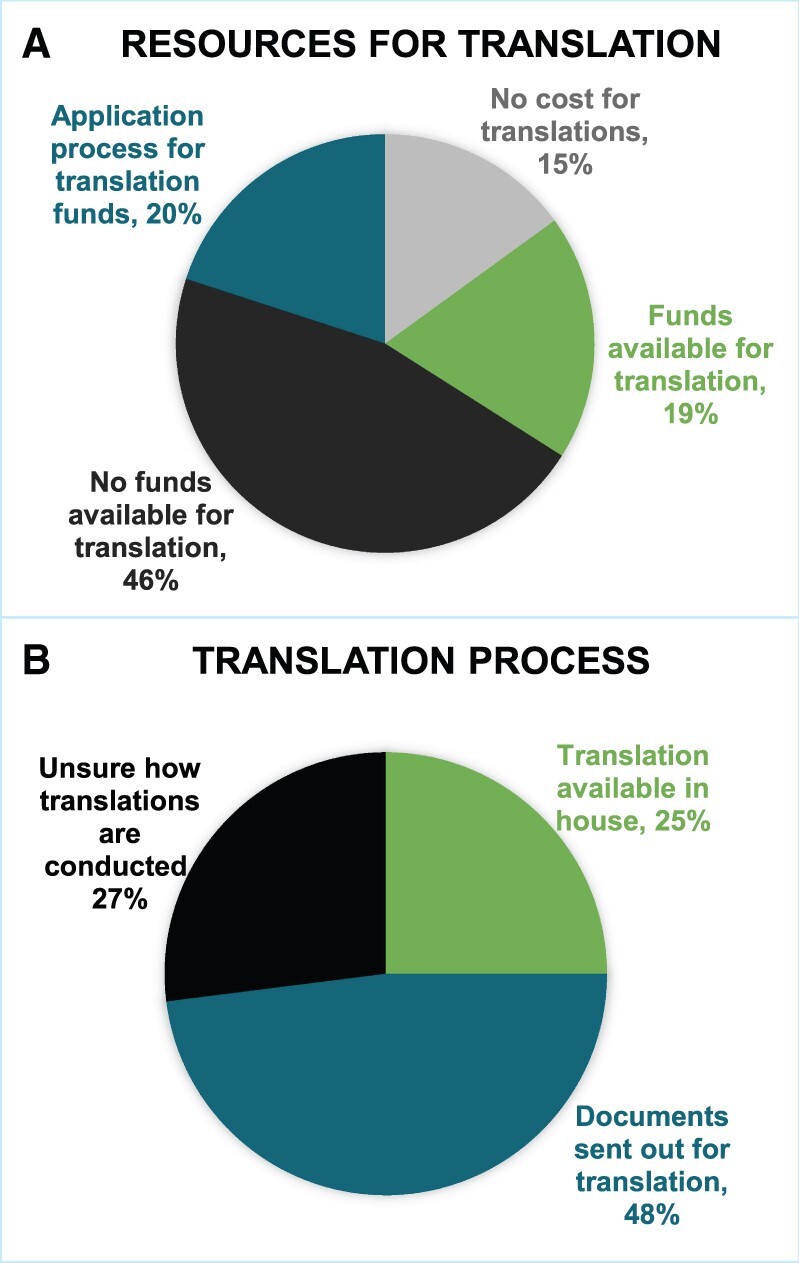
Available resources for translation of consent documents (**A**) and the translation process (**B**).

Half (69/139) of IRBs required consent forms to be translated for participants who speak languages other than English to enable them to enroll in the clinical trial; 17% (23/139) of respondents were not sure if there were translation requirements at their centers. Among those centers with requirements, in 49% (34/69) of institutions, translated consent forms depended on anticipated accrual of participants who speak languages other than English based on local demographics, and 29% (20/69) were required to have a translated consent form regardless of projected accrual of participants who speak languages other than English. Costs for translation were covered at 19% (26/139) of the institutions, whereas others had a process to apply for funds (28/139 [20%]) (Figure 2A). Forty-six percent (64/139) of institutions did not have funds available to cover the costs of translation. Translation services were available on-site at 24% of institutions (34/139), whereas 48% (67/139) needed to send translation requests to an outsourced translation service (Figure 2B). The remaining 27% (n = 38) were unsure of how translation services were accessed.

In total, 58% of institutions (81/139) always or sometimes used centrally translated consent forms provided by the NCI Central IRB, but 12% (17/139) reported that their local IRB would not allow use of these translated forms, and 6% (9/139) were unaware of these translated consent forms (Table 2). Nonacademic institutions more often reported always using Central IRB–translated consent forms when available than academic institutions did (62% vs 38%, *P* = .02).

**Table 2.** Regulatory requirements for gaining consent from participants who speak languages other than English (N = 139), by institution size, institution type, and role of the respondent

		Institution size			Institution type			Respondent role		
	Total, No. (%)	Small institution (n = 99)	Large Institution (n = 40)	*P*	Academic institution (n = 100)	Nonacademic institution (n = 39)	*P*	**Other** ^a^ **(n = 59)**	Physician (n = 79)	*P*
Short-form consent forms available										
Yes	83 (60)	53 (54)	30 (75)	.02	61 (61)	22 (56)	.57	39 (66)	43 (54)	.19
No	55 (40)	45 (45)	10 (25)		38 (38)	17 (44)		20 (44)	35 (45)	
Missing	1 (<1)	1 (1)	0 (0)		1 (1)	0 (0)		0 (0)	1 (1)	
Among institutions that use a short-form consent form (n = 83)										
No. of languages available in the short-form consent form				.28			.002			.03
≤10	48 (58)	33 (62)	15 (50)		29 (48)	19 (86)		18 (46)	30 (70)	
>10	35 (42)	20 (38)	15 (50)		32 (53)	3 (14)		21 (54)	13 (30)	
Comfort using short-form consent form				.64			>.99			.016
Comfortable	71 (93)	43 (81)	28 (93)		53 (87)	18 (82)		29 (74)	41 (95)	
Uncomfortable	5 (7)	4 (8)	1 (3)		4 (6)	1 (5)		5 (13)	0 (0)	
Missing	0 (0)	6 (11)	1 (3)		4 (6)	3 (14)		5 (13)	2 (5)	
Never used short-form consent form	7 (8)	—	—		—	—		—	—	
Percentage of speakers of languages other than English who provided consent using the short-form consent form				.19			.16			.31
None	7 (8)	6 (11)	1 (3)		5 (8)	2 (9)		4 (10)	3 (6)	
1%-50%	38 (46)	26 (49)	12 (40)		24 (39)	14 (64)		21 (54)	17 (40)	
≥51%	35 (42)	19 (36)	16 (53)		29 (48)	6 (27)		13 (33)	21 (49)	
Missing	3 (4)	2 (4)	1 (3)		3 (5)	0 (0)		1 (3)	2 (5)	
IRB requires translation of consent documents										
Yes	69 (50)	50 (51)	19 (48)	.31	56 (56)	13 (33)	.04	35 (59)	34 (43)	.13
No	46 (33)	30 (30)	16 (40)		30 (30)	16 (41)		15 (25)	30 (38)	
Not sure	23 (16)	19 (19)	4 (10)		13 (13)	10 (26)		8 (13)	15 (19)	
Missing	1 (1)	0 (0)	1 (2)		1 (1)	0 (0)		1 (3)	0 (0)	
Among institutions where the institutional review board requires translation of consent forms (n = 69)										
IRB requirements for use of translated consent documents				.23			.61			.026
For protocol with a certain percentage of patients from specific backgrounds	34 (49)	24 (48)	10 (53)		26 (46)	8 (62)		13 (37)	21 (62)	
Any protocol with participants who speak languages other than English	20 (29)	17 (34)	3 (16)		17 (30)	3 (23)		10 (29)	10 (29)	
Other	15 (22)	9 (18)	6 (32)		13 (23)	2 (15)		12 (34)	3 (9)	
Institution allows Central IRB–approved translated consent documents										
Always	61 (44)	49 (49)	12 (30)	.06	37 (38)	24 (62)	.02	27 (46)	34 (43)	.22
Sometimes	20 (14)	15 (15)	5 (13)		16 (16)	4 (10)		8 (14)	11 (14)	
IRB does not allow these translations	17 (12)	12 (12)	5 (13)		16 (16)	1 (93)		7 (12)	10 (13)	
Not aware that these translations exist	9 (6)	7 (7)	2 (5)		5 (5)	4 (10)		1 (2)	8 (10)	
Other	30 (22)	15 (15)	15 (38)		24 (24)	6 (15)		16 (27)	14 (18)	
Missing	2 (1)	1 (1)	1 (1)		2 (2)	0 (0)		0 (0)	2 (2)	
Requirement that in-person interpreters be used										
Yes	61 (44)	40 (40)	21 (53)	.34	44 (44)	17 (44)	.24	25 (42)	36 (46)	.93
No	71 (51)	53 (54)	18 (45)		53 (53)	18 (46)		31 (53)	39 (49)	
Not sure	7 (5)	6 (6)	1 (3)		3 (3)	4 (10)		3 (5)	4 (5)	

a Other: CRA, Nurse, NCORP Administrator. IRB = institutional review board.

Short-form consent documents were used for participants who speak languages other than English at 60% of institutions (83/139). Among these 83 institutions, 42% (n = 35) had short forms available in more than 10 languages, and 42% (n = 35) reported obtaining consent from at least half of the participants who speak languages other than English using a short form (Table 2). Large institutions more often used short forms than did small institutions (75% vs 54%, *P* = .02). Of the 83 institutions that used short forms, 85% (n = 71) were comfortable using short forms.

### Availability of interpreter and language-concordant services

In total, 81% (113/139) of institutions had access to trained medical interpreters, and 77% (107/139) reported access to in-person interpreter services (Table 3). Large institutions more often had access to in-person interpreter services than did small institutions (94% vs 71%, *P* < .01). Fifty-one percent (71/139) of institutions reported that more than 10% of their oncology clinicians were bilingual or multilingual. Among institutions with at least 1% multilingual provider, thirty percent (n = 32) endorsed having a process for multilingual clinician certification. In addition, 57% (n = 79) reported that their team (research or clinical) included bilingual staff, and 57% of these 79 (n = 44) had a certification process for bilingual staff; noncertified interpreters were rarely or never used (90% [n = 125]). Regarding regulatory requirements for interpretation during informed consent discussions, 44% (61/139) of institutions were required to use in-person interpreters.

**Table 3.** Availability of interpretation and language-concordant services (N = 139)

Characteristic	No. (%)
Specifically trained medical interpreters available	
Yes	113 (81)
No	17 (12)
Unsure	7 (5)
Access to medical interpreter services^a^	
In-person interpreters available	107 (77)
Interpreter services available through on-demand application	86 (62)
Telephone interpreter services available	76 (55)
No interpreter services available	0 (0)
Certification process for bilingual or multilingual clinicians (n = 109)	
Yes	32 (29)
No	53 (49)
Unsure	22 (20)
Clinical or research staff who are bilingual	
Yes	79 (57)
No	51 (37)
Unsure	5 (4)
Certification process for bilingual staff (n = 77)	
Yes	44 (57)
No	18 (23)
Unsure	15 (20)
How often are noncertified interpreters used for consent?	
Always	0 (0)
Often	12 (9)
Rarely	57 (41)
Never	68 (49)
In-person interpreters required for informed clinical trial consent from patients speaking languages other than English	
Yes	61 (44)
No	71 (51)
Unsure	7 (5)
Perceived difficulty in obtaining consent from patients speaking languages other than English for clinical trials	
Very difficult	11 (8)
Somewhat difficult	54 (39)
Not too difficult	39 (28)
Not difficult at all	34 (25)

a Numbers may not sum to 100% because of an option to select all that apply.

### Perceptions of consenting participants or surrogate decision makers who speak languages other than English

**Figure 3.**
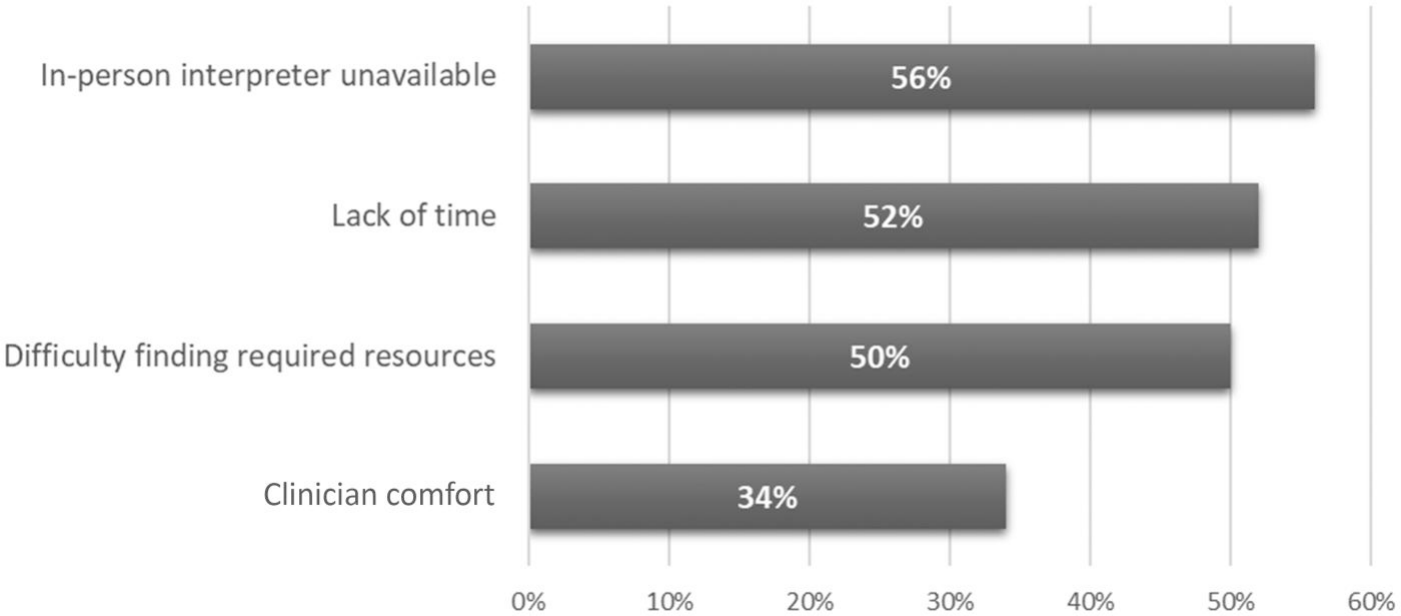
Factors contributing to difficulty in obtaining consent from participants who speak languages other than English.^a^ ^a^ Respondents were allowed to select more than 1 answer; percentages may not sum to 100%. Represents 104 institutions that endorsed some level of difficulty gaining consent from participants who speak languages other than English to participate in clinical trials.

Seventy-five percent (104/139) of institutions reported some level of difficulty in gaining consent from participants who speak languages other than English, with 47% (65/139) rating gaining consent “somewhat difficult” or “very difficult” (Table 3). Among the institutions that reported some level of difficulty, the most commonly endorsed barriers to obtaining consent were lack of available in-person interpreters (56% [58/104]), time (52% [54/104]), required institutional resources (50% [52/104]), and clinician comfort (34% [35/104]) (Figure 3). Overall, most (83% [116/139]) reported that lack of interpreter services did not affect their ability to enroll participants who speak languages other than English, but when we examined responses from the 65 institutions that reported a “somewhat” or “very” difficult time gaining consent from participants who speak languages other than English, this proportion decreased from 85% to 72% (47/65).

## Discussion

This study assessed institutional capacity, resources, and barriers to inclusion of participants or surrogate decision makers who speak languages other than English in pediatric cancer clinical trials. We found that although half of IRBs require translated consent forms, most COG institutions also use short-form consent documents, highlighting the multiple layers of regulatory resources required to offer inclusive approaches to enroll participants who speak languages other than English in cancer clinical trials. In addition, although interpreter services are often available, in-person interpreter requirements from the IRB may impede institutions, particularly small institutions, from equitably enrolling participants who speak languages other than English in clinical trials. Overall, our findings demonstrate that current processes and resources at many COG-affiliated institutions may impair equitable access to clinical trials and contribute to the underrepresentation of persons who speak languages other than English in pediatric cancer clinical trials (22).

Regulatory barriers were reported regarding consent form requirements for enrollment of participants who speak languages other than English. A tension existed between the regulatory requirements to ensure availability of translated consent forms and the allowance of or restrictions on using short forms. Translation service requirements have historically been expensive and time-consuming, delaying the opportunity to enroll persons who speak languages other than English in a trial (31). Translating consent forms is a multistep process that tends to require costs that are rarely covered in research budgets. In our survey, nearly half of institutions did not have access to translation cost coverage, potentially rendering it impossible to offer clinical trials to populations that speak languages other than English. Recently, NCI-sponsored protocols facilitated translating consent forms into Spanish to alleviate institutional barriers to translation or short-form use, but this service does not uniformly cover translation of all study materials (eg, surveys, ancillary studies, study reports [results] and assessments), and in our study, some institutions—more often academic rather than nonacademic institutions—reported restrictions related to using centrally translated documents. This finding is concerning, especially given that prior research has demonstrated that clinicians consider a lack of adequately translated consent forms as a major barrier to enrolling adults with cancer who speak languages other than English (32).

In their current form, short-form consent documents are inadequate for communicating the purpose of the research or the details of participation (27). The inability to use these documents at 40% of institutions in our study, however, likely further hinders recruitment of persons who speak languages other than English. Current consent documents are often lengthy, intimidating, and difficult to digest, affecting comprehension of informed consent and decision-making abilities, particularly in individuals with low health literacy or individuals who speak languages other than English (33,34). An abbreviated document, such as a visual aid, may be more effective in communicating complex information and may offer flexibility for research participants, similar to telehealth or virtual consent allowances (35,36). In prior research, potential participants for a clinical trial more thoroughly read a shortened document with illustrations than they did a full consent document, regardless of primary language (37). Our findings support the need to continue the broader discussion of how to balance time and attention constraints with provision of comprehensive study information (38,39).

Additional investment, either from the NCI, institutions, foundations, companies, or other study sponsors, should be allocated to ensure that translated consent forms and study documents are available and acceptable for use to support institutions in activating clinical trials and addressing regulatory barriers. The Belmont Report encouraged equitable selection of study participants and mandated autonomy in the informed consent process (40). To ensure autonomy and to avoid excluding individuals who speak languages other than English, institutions should ideally have translated research study documents to match the languages spoken by the patients in their catchment area. For institutions where a high proportion of patients speak Spanish or another common language, efforts should be made to have consents forms, surveys, and related study materials available in that language whenever possible.

Availability of high-quality, professional medical interpreters is critical for clinical trial informed consent (41). In our study, most institutions had access to medical interpreter services through a combination of in-person, online, and telephone-based services. Even with access to interpreter services, however, lack of in-person interpreters, a requirement for gaining consent from participants who speak languages other than English at nearly half of institutions, was the most frequently cited barrier to recruiting these participants. This finding suggests that even with access to interpreter services, additional barriers, including regulatory requirements for in-person interpretation and practical challenges coordinating interpreter availability in a busy clinic or inpatient setting, where consent discussions often occur, remain a challenge. This finding is consistent with a qualitative study of pediatric oncology clinicians, where 1 of the top barriers to enrolling persons who speak languages other than English was clinician-patient language discordance (42). Lack of in-person interpreters may lead to situations where the clinicians or patient and family do not feel sufficiently supported to discuss or receive information about clinical trials. Moreover, a suboptimal discussion may ensue that leads to a diminished patient-clinician therapeutic alliance (43). A 2006 survey of clinicians, parents, and interpreters identified similar concerns, noting that availability of interpreters as well as using simpler, easy-to-understand language were necessary to reduce barriers to care (44). Having readily available, trained professional interpreters improved the informed consent process in a study of hospitalized patients undergoing invasive surgical procedures (45). Pediatric oncology settings are similarly complex, with initial treatment options often presented to the family in an acute, inpatient setting. Because linguistically concordant health care may improve access to and engagement with the health-care system and, by extension, cancer therapy (46), additional training may be warranted to ensure that high-quality consent discussions are feasible.

In pediatric oncology, barriers and facilitators of communication have been well described for English-speaking families (47). Recent studies have outlined potential strategies to improve clinical trial participation, including optimizing the research infrastructure at an institution, developing and testing shared decision-making interventions, and provision of additional psychosocial support to people considering participation for themselves or a family member (48,49). The next critical step is to apply—or adapt, if necessary—these innovative strategies to focus on pediatric patients and their parents who speak languages other than English as they consider treatment options for cancer, including participation in a clinical trial.

Our data suggest that most institutions have access to some resources, including interpreter services or translated consent documents; these resources can be expanded upon or adapted to ensure consistent access to and offering of clinical trials. Certainly, the culture of pediatric oncology is an exemplar of clinical trial participation and success, as past decades have shown, and has led to current successes in many pediatric cancer treatments to date (50,51). A recent study, however, showed an overall decline in participation in COG clinical trials over time (24); thus, efforts aimed at improving communication with potential participants about research trials are urgently needed.

Dedicated, multilevel interventions are needed to address current disparities in access to high-quality health care, including clinical trials (17,47,52,53). Rethinking our current informed consent process, which uses lengthy, complicated, and often overwhelming documents, should be considered regardless of language spoken (38,39). In addition to the availability of translated consent forms, the health-care system should prioritize designated time and space for long discussions, training in cultural competence to reduce clinician discomfort while gaining consent from participants who speak languages other than English, and access to high-quality interpreter services that represent the languages most commonly spoken among local patients (54). Without these resources, addressing clinical trial underrepresentation will be difficult. In addition, institutions should ensure that all available resources to support diverse enrollment within their local infrastructure or centrally are used; the ability to use Central IRB–translated consent documents or access translation services should be prioritized, as well. Another example is the integration of patient navigators, a valuable resource that may increase access to, understanding of, and consent to participate in cancer clinical trials (46,52). Depending on the local context of and persons an institution serves, attention should be given to hiring staff who can provide linguistically concordant care; training procedures for multilingual staff and clinicians might also be shared across institutions to reduce resource burdens (55). The implications from our study are not only relevant for the pediatric oncology community but more broadly across cancer care settings, where inclusion in clinical trials and generalizability of findings are critical (56).

We used an interdisciplinary team to develop our survey instrument, using previously established modes of survey distribution to reach diverse and geographically distributed COG-affiliated institutions across the United States, Canada, and Australia. Our study has some limitations, however, including the potential for nonresponse or recall difficulties. As this was a voluntary survey study without full participation from all COG-affiliated institutions, the results may not be fully representative of the COG consortia and do not represent the experiences of institutions not affiliated with COG. In addition, the responses relied on the knowledge of the lead clinical research associate or COG principal investigator and represent only the perspective of those individuals delivering care, not those individuals receiving it. Further, we did not survey medical interpreters, IRB staff, or other administrators, limiting the scope of understanding of institutional and regulatory barriers. Moreover, we did not conduct an evaluation of costs associated with interpretation and translation services, which could help develop future interventions and policy. Some institutions may serve a larger population of people who speak languages other than English or where a language other than English is the dominant language; however, most institutions represent geographic locations where English is the most common language. Finally, the landscape of language equity, communication, language preference, and therapeutic misconception is rapidly evolving. Although we did not use a validated instrument as one does not currently exist to assess this specific topic, the survey was developed with this in mind and used multidisciplinary expertise to provide insight into a complex issue. This study highlights actionable barriers to gaining consent from persons who speak languages other than English and will inform areas for further investigation and interventions to provide equitable informed consent processes for all children with cancer and their families.

COG-affiliated institutions face multiple resource-specific challenges that create barriers to recruitment and enrollment of participants or surrogate decision makers who speak languages other than English in cancer clinical trials. Our findings offer directions to focus future strategic efforts to reduce recruitment barriers to COG clinical trials and ensure equitable clinical trial representation and improved outcomes for all.

## Supplementary Material

pkae047_Supplementary_Data

## Data Availability

All data referenced in this publication are available upon reasonable request.
