# Supplementary material for: Clinical trial recruitment of people who speak languages other than English: a Children’s Oncology Group report
Source: JNCI Cancer Spectr. 2024 Jun 18;8(4):pkae047. doi: 10.1093/jncics/pkae047 (PMC11272047; doi:10.1093/jncics/pkae047)
Supplement: pkae047_Supplementary_Data [file pkae047_supplementary_data.docx]

**Supplementary Material**

**Supplementary Methods: Survey Instrument**

**Diversity and Health Disparities: Language Equity Working Group**

***Survey request 2022***

**Survey Title:** Assessment of Language and Consent Processes at COG Institutions

**Question 1:**

What is your role at your institution?

1. Physician
2. Nurse Practitioner/Advanced Practice Provider
3. Nurse (RN)
4. Administrator
5. Other (please specify):______

**Question 2:**

What proportion of providers in your pediatric oncology division are bi/multilingual?

1. None
2. 1 – 5%
3. 10 – 25%
4. More than 25%

If 2, 3, or 4, Branching: Are those who are multilingual certified by your institution?

1. Yes, all.

2. Yes, some.

3. No

4. I don’t know

**Question 3:**

In which country is your institution located? ______(select from dropdown, US, Canada, Australia, ?others)

If US or Canada, what is your institutional zip/postal code _____

**Question 4:**

Which of the following best describes your institution?

1. Academic medical center
2. Non-academic community or public hospital
3. Non-academic private hospital
4. Military hospital
5. Other: please specify

**Question 5:**

How many newly-diagnosed pediatric oncology patients does your institution see per year?

1. <50
2. 50 – 100
3. 101 – 125
4. 126 – 150
5. >150

**Questions 6:**

Please select and rank the top three languages, other than English, spoken by your patients and their families?

1. Arabic
2. Chinese (Mandarin, Cantonese, or other)
3. French
4. German
5. Korean
6. Russian
7. Spanish
8. Tagalog
9. Vietnamese
10. Other: __________________

**Question 7:**

Does your institution have a library of short-form consent documents to facilitate consenting non-English preferring persons?

1. Yes – if yes, how many languages do you have available? (1 – 2 , 3 – 5, 6 – 10, >10)
2. No

**Question 8:**

How comfortable are you consenting persons with LEP using a short-form consent?

1. Very comfortable
2. Somewhat comfortable
3. Uncomfortable
4. I’ve never done this

**Question 9:**

What percentage of non-English participants are consented using a short-form consent document in place of a full translated consent document?

1. None of the non-English consents are conducted with short-form consents (All non-English consents obtained with translated full consents).
2. About 25% of non-English consents are conducted with short-form consents
3. About 50% of non-English consents are conducted with short-form consents
4. About 75% of non-English consents are conducted with short-form consents
5. All non-English consents are conducted with short-form consents

**Question 10:**

Does your institutional review board (IRB) have requirements for consent form documents to be translated?

1. Yes (please see next question for additional information about the requirements)
2. No, our institution does not have any requirements for translation of consent forms
3. I am not sure

If yes, which of the following most closely match your IRB’s requirements?

- 1. Our institution requires that any protocol that will enroll a certain proportion of people from specific backgrounds (e.g., Hispanic/Latinx) must provide consent forms in a language other than English (e.g., Spanish).
  2. Our institution requires that any protocol which allows participation by persons with LEP must provide consent forms in that language.
  3. Other (please describe)____.

**Question 11:**

When you need translation of a clinical trial consent form, which of the following best describes the process and resources available to obtain translation? (Select one for each section)

1. There is no cost to us for translation (e.g. institutional translation support)
2. We have funds readily available to cover translation costs
3. We have a process to apply for funds to cover translation costs
4. We do not have funds available to cover translation costs

Regarding process:

1. We have in-house translation available
2. We send documents out for translation
3. I’m not sure how we obtain translation of consent forms

**Question 12:**

Is your institution affiliated with a Clinical Translational Science Award (CTSA) Program?

1. Yes
2. No

If yes:

1. Yes, and we partner with our CTSA to translate clinical research IRB documents
2. Yes, but I am not sure if we partner with our CTSA for translation
3. Yes, but we do not partner with our CTSA for translation

**Questions 13:**

How often do you utilize the Central-IRB (CIRB)-approved translated consent form and other study documents, when available for a COG study?

1. We always use these translations and submit through our IRB after modifying with local boilerplate language
2. We sometimes use these translations
3. Our IRB does not allow these translations
4. We were not aware that these translations exist
5. Other (please specify):

**Question 14:**

Does your institution require that in-person medical interpreters are utilized to obtain informed consent for persons with LEP?

1. Yes
2. No
3. I’m not sure

If no, (Branching): Are providers consenting with phone interpretation services (yes/no) or using family members that are not medical interpreters (yes/no)?

**Question 15:**

To your knowledge, how much difficulty do providers at your institution have obtaining parental permission or consent from persons with limited English proficiency (LEP) for clinical trials?

1. It is very difficult
2. It is somewhat difficult
3. It is not too difficult
4. Not difficult at all – we have an organized system to support it

If 1, 2, or 3:

What factors contribute to this difficulty? (Choose all that apply)

- provider comfort
- lack of time
- difficulty finding the resources needed (short form, translated consent form)
- In-person medical interpreter unavailable
- Other (please specify): _____

**Question 16:**

In your hospital’s clinical settings (inpatient, outpatient), do you have access to medical interpreter services? Please select all that apply.

1. Yes, we have in-person medical interpreters available
2. Yes, we have interpreter services through an on-demand application (e.g. Boostlingo, Martti)
3. Yes, we have interpreter services through a telephone service
4. No, our institution does not provide interpreter services

IF yes, “How often does availability of needed interpreter services impact your ability to enroll patients onto COG trials?”

1. Always
2. Often
3. Rarely
4. Never

IF yes, “How often does quality of needed interpreter services impact your ability to enroll patients onto COG trials?”

1. Always
2. Often
3. Rarely
4. Never

**Question 17:**

To which of the following does your pediatric oncology division have access:

- Specifically-trained medical interpreters (yes/no/unsure)
- Bilingual research coordinators (yes/no/unsure)
- Use of staff who are bilingual (yes/no/unsure
- If yes, do you have a process for certification of bilingual staff (yes/no/unsure
- Other language services/resources (yes/no/unsure)

**Question 18:**

How often have you used non-certified individuals (staff, family members, etc.) to assist in a consent discussion?

1. Always
2. Often
3. Rarely
4. Never

**Question 19:**

In your hospital’s clinical settings (inpatient, outpatient pediatric oncology), do you routinely screen patients or caregivers for literacy (e.g., education level) or health literacy (validated assessment) levels?

1. Yes, (branching)
   1. We routinely assess literacy levels during our initial consultation
      1. Who conducts the assessment?
         1. Physician
         2. Social worker
         3. Nurse
         4. Other (please specify):____
   2. We use assessment tools
      1. SDOH
      2. PAT
      3. NVS
      4. Institutional specific survey
      5. Other tool (please specify):____
2. No, we do not routinely assess literacy/health literacy levels

**Question 20:**

Does your institution use any of the following to deliver information/educate your patients or caregivers? Please select all that apply.

1. Yes, we use infographics embedded in educational materials
2. Yes, we provide families with the COG patient-family handbook or other guide at diagnosis
3. Yes, we assist families in accessing and setting up the KidsCare app
4. Yes, we do something else (please specify):______
5. No
6. I am not sure

**Question 21: (open-ended section)**

What is the biggest barrier to enrolling children of parents with LEP or adult participants (18 years and older) with LEP on clinical trials at your institution? _____

What ideas do you have for how COG can improve the processes for enrolling children of parents with LEP or adult participants with LEP on COG clinical trials? _______

We are interested in conducting focus groups or individual interviews to better understand provider experiences with language barriers to clinical trial enrollment and retention. If you would be willing to participate in one of these groups, please provide your email here: _____

**Supplementary Table 1:** **Definitions for Language Equity concepts**

| **Term/Phrase** | **Definition** |
| --- | --- |
| **Translation** | Rendering written content presented in one language (the source language) into another language (the target language) |
| **Interpretation** | Rendering spoken content presented in a source language into a target language in real time |
| **Limited English Proficiency (LEP)** | Individuals who do not speak English as their primary language and who have limited ability to read, speak, write or understand English can be limited English proficient, or “LEP” |
| **Non-English Language Preferring (NELP)** | Individuals who prefer a non-English language with respect to a particular type of service, benefit, or encounter |
| **Language Other than English (LOE)** | Similar to NELP, but preferred for health-related discussions. This eliminates the concept of “preference,” since for many individuals communicating in a specific language is a necessity, not a choice |
